# The clinical complication scale of Fondazione Don Gnocchi for classifying clinical complications in patients with severe acquired brain injury: development and multicenter validation

**DOI:** 10.3389/fneur.2025.1537093

**Published:** 2025-03-12

**Authors:** Anna Estraneo, Maria Rosaria Fiorentino, Tommaso Cibellis, Biagio Campana, Pietro Balbi, Valentina Carli, Elena Vatteroni, Guya Devalle, Francesco Mantelli, Mattia Villa, Alessandra Bianchi, Maria Concetta Costa, Marina Rossi, Angela Comanducci, Jorge Navarro, Alessandro Viganò, Agnese De Nisco, Francesca Draghi, Bahia Hakiki, Alfonso Magliacano

**Affiliations:** ^1^IRCCS Fondazione Don Carlo Gnocchi ONLUS, Florence, Italy; ^2^Polo Specialistico Riabilitativo, Fondazione Don Carlo Gnocchi ONLUS, Sant’Angelo dei Lombardi, Italy; ^3^Centro S. Maria dei Poveri, Polo Riabilitativo del Levante Ligure Fondazione Don Carlo Gnocchi ONLUS, La Spezia, Italy; ^4^Istituto Palazzolo Fondazione Don Carlo Gnocchi ONLUS, Milan, Italy; ^5^Centro Spalenza Fondazione Don Carlo Gnocchi ONLUS, Rovato, Italy; ^6^IRCCS SM Nascente Fondazione Don Carlo Gnocchi ONLUS, Milan, Italy; ^7^Department of Experimental and Clinical Medicine, University of Florence, Florence, Italy

**Keywords:** severe acquired brain injury, clinical complications, consciousness disorders, rehabilitation, outcome

## Abstract

**Introduction:**

Patients with severe acquired brain injury have a high risk of developing clinical complications that affect clinical outcome and rehabilitation program. Early identification of clinical complications would allow to treat them appropriately and to prevent their worsening. However, available clinical scales for recording clinical complications are not appropriately tailored for this population. The present multicenter study aimed at developing and validating a new scale to categorize the clinical complications: the Clinical Complication Scale of the Fondazione Don Gnocchi (FDG-CCS).

**Methods:**

Six Intensive Neurorehabilitation Units enrolled consecutively admitted patients with severe brain injury. Demographic, anamnestic, and clinical data were collected at study entry. For each enrolled patient, two independent examiners (A and B) administered the FDG-CCS considering 2 weeks as an observation time window. Concurrently, a third examiner (C) administered the Comorbidities Coma Scale. The blinded examinations were analyzed to assess the inter-rater agreement (A vs. B) and the concurrent validity of the FDG-CCS with respect to the Comorbidities Coma Scale (C).

**Results:**

A total of 42 patients (22 patients with and 20 emerged from prolonged disorder of consciousness) were enrolled. The FDG-CCS total score did not differ in the two subgroups of patients. Metabolic (examiner A = 33%; examiner B = 43%), gastro-intestinal (A = 31%; B = 26%), cardio-vascular (A = 26%; B = 29%), respiratory (A = 21%; B = 21%), and musculo-skeletal disorders (A = 19%; B = 14%) were the most frequent complications. Inter-rater agreement for the total score of the FDG-CCS resulted to be good (intra-class correlation coefficient = 0.865; *p* < 0.05), and the FDG-CCS total score correlated significantly with the total score of the Comorbidities Coma Scale (A, *ρ* = 0.356; *p* = 0.01; B, ρ = 0.317; *p* = 0.02).

**Discussion:**

The present multicenter study proposed and validated a novel clinical tool for the categorization of clinical complications of patients with severe brain injury. This clinical tool could help the rehabilitation team for planning tailored treatment and prevention of clinical complications that negatively impact patients’ outcomes and hamper rehabilitation programs.

## 1 Introduction

Patients with severe Acquired Brain Injury (sABI) show high clinical complexity because of coexisting cognitive-motor disability, with high burden of specialized care and dependence for daily life activities (1, 2). Additionally, these patients have a high risk of developing clinical complications that can lead to a high occurrence of re-hospitalization in acute care wards (3) and makes the post-acute rehabilitation treatment difficult (1). Some patients with sABI can evolve from the comatose state to the prolonged Disorders of Consciousness (pDoC), which include patients in Vegetative State/Unresponsive Wakefulness Syndrome (VS/UWS; i.e., awake patients, but no evidence of conscious behaviors) (4), and patients in Minimally Conscious State (MCS; i.e., patients with minimal but reproducible intentional behaviors) (5). For patients with sABI and pDoC, the most frequent clinical complications include epilepsy, respiratory and genito-urinary infections, bedsores, hypertonia, heterotopic ossification, deep vein thrombosis, cardiac and endocrine-metabolic dysfunctions (6–9). Some of these clinical complications negatively impact survival (e.g., metabolic disorders), recovery of consciousness (i.e., epilepsy) (6, 10) and recovery of motor disability (11). Based on this evidence, the American Academy Neurology guidelines strongly recommended identifying clinical complications, in order to treat them appropriately and to prevent their worsening (12).

The available clinical scales for recording clinical complications have been specifically developed for other patients’ populations, such as frail elderly or cancer patients (e.g., Cumulative Illness Rating Scale, CIRS; Greenfield scale) (13, 14). However, these scales can neglect the more typical and frequent conditions for patients with sABI, that hamper the rehabilitation and the recovery of motor abilities such as paroxysmal sympathetic hyperactivity or heterotopic ossifications (11, 15, 16). Currently, only one scale has been specifically designed for the registration of clinical complications in the population of patients with pDoC: the Comorbidities Coma Scale (CoCoS) (17). Although very detailed, the CoCoS fails to record some specific dysfunction (e.g., heterotopic ossification) and provides a different classification of severity for each disease, which makes it complex to compile the scale in the routine of clinical practice.

In this context, the present multicenter study aimed at developing and validating a novel scale to record the clinical complications in patients with sABI (i.e., the Clinical Complication Scale of the Fondazione Don Gnocchi, FDG-CCS).

## 2 Materials and methods

### 2.1 Study design

The study was conducted in three distinct phases, which involved professionals from six Intensive Neurorehabilitation Units, qualified by the Italian National Health System for the care of post-acute patients with sABI. The participating centers are part of the Department for rehabilitation of patients with sABI at Fondazione Don Gnocchi ONLUS (Italy).^1^

*In the first phase*, clinicians with high expertise in the management of the post-acute phase of sABI developed the final version of FDG-CCS. The FDG-CCS was elaborated based on a previous longitudinal study that aimed to record the presence of the most frequent clinical complications in patients with sABI and their impact to patients’ outcomes (6). In the Estraneo et al.’s study (6), clinical complications were grouped in 10 categories. The first six categories, modeled on the CIRS (18), included the clinical complications affecting the main systems of the body (i.e., endocrine-metabolic, cardio-vascular, musculo-skeletal-cutaneous, gastro-intestinal, genito-urinary tract, respiratory). Four additional categories included clinical conditions that more frequently and specifically occur in patients with sABI (i.e., neurosurgical complications, epilepsy/myoclonus, heterotopic ossification, and paroxysmal sympathetic hyperactivity). The severity of clinical complications was classified as moderate or severe on the basis of the intensity of the treatment protocol required (in categories 1–8); severity of heterotopic ossification (in category 9) was related to the number of affected joints; paroxysmal sympathetic hyperactivity (category 10) was only classified as present or absent (see Table 1).

**Table 1:** Comparison between the original checklist (6) and the FDG-CCS.

Original checklist	FDG-CCS	Specific complication	Score	
Endocrine-metabolic	1. Metabolic	Electrolyte imbalances, anemia, hypoalbuminemia, malnutrition, other	Absent	0
			Mild	1
			Moderate	2
			Severe	3
	Endocrine	Dysthyroidism, hypopituitarism, diabetes mellitus, diabetes insipidus, SIADH, central salt wasting syndrome, adrenocortical insufficiency, other	Absent	0
			Mild	1
			Moderate	2
			Severe	3
2. Cardio-vascular	Cardio-vascular	Heart failure with preserved or reduced ejection fraction, acute or chronic myocardial ischemia, acute or chronic arrhythmia, arteriovenous thrombosis, other	Absent	0
			Mild	1
			Moderate	2
			Severe	3
3. Musculo-skeletal-cutaneous	Musculo-skeletal	Spasticity, fractures, muscle injuries, tendon injuries, tendon retractions, other	Absent	0
			Mild	1
			Moderate	2
			Severe	3
	Cutaneous	Bed sores, wounds, skin accesses, other	Absent	0
			Mild	1
			Moderate	2
			Severe	3
4. Gastro-intestinal	Gastro-intestinal	Bleeding, ulcer, intestinal obstruction or paralysis, peritonitis, clostridium difficile enteritis, non-specific diarrhea, intestinal infarction, cholelithiasis, hepatitis, pancreatitis, other	Absent	0
			Mild	1
			Moderate	2
			Severe	3
5. Genito-urinary tract	Genito-urinary tract	Infections, bleeding, urolithiasis, urinary tract obstructions, acute or chronic renal failure, other	Absent	0
			Mild	1
			Moderate	2
			Severe	3
6. Respiratory	Respiratory	Pneumonia, COPD, respiratory insufficiency, tracheomalacia, tracheal stenosis, tracheo-esophageal fistula, other	Absent	0
			Mild	1
			Moderate	2
			Severe	3
7. Neurological/Neurosurgical	Neurosurgical complications	Hydrocephalus, new brain injury, ventriculo-peritoneal shunt dysfunction, sinking skin flap syndrome, other	Absent	0
			Mild	1
			Moderate	2
			Severe	3
8. Epilepsy/myoclonus	Epilepsy/myoclonus	Generalized or partial seizures, generalized or partial myoclonus, convulsive status epilepticus, non-convulsive status epilepticus, other	Absent	0
			Mild	1
			Moderate	2
			Severe	3
9. HO	HO	Specify location involved	Absent	0
			Mild	1
			Moderate	2
			Severe	3
10. PSH	PSH	Specify semiology	Absent	0
			Occasional	1
			Frequent	2
			Persistent	3
/	Sepsis	Specify system/apparatus or organ of origin (if identified)	Absent	0
			Present	1

For the detailed version of the FDG-CCS and its scoring system (see Supplementary material). Severity levels for the original checklist (6). Groups 1–8 were classified as moderate (i.e., requiring acute or chronic pharmacologic treatment not associated with intensive clinical monitoring), or severe (i.e., requiring urgent and/or continuous or sub-continuous treatment and clinical monitoring or surgical intervention). Group 9 was classified as moderate (i.e., only 1 joint was involved), or severe, (i.e., 2 or more joints were involved or when range of motion of the affected joints was drastically reduced). Group 10 was not assessed, but we only considered the presence of acute onset of paroxysmal episodes. COPD, Chronic Obstructive Pulmonary Disease; HO, Heterotopic Ossifications; PSH, Paroxysmal Sympathetic Hyperactivity; SIADH, Syndrome of Inappropriate secretion of AntiDiuretic Hormone.

The FDG-CCS differed from the previous version of the checklist because it included 13 categories of clinical complications and introduced a numeric rating system. The two items “endocrine-metabolic disorders” and “musculo-skeletal-cutaneous complications” of the previous checklist were split in four separate categories (i.e., endocrine disorders, metabolic disturbances, musculo-skeletal and cutaneous complications), to assess their occurrence more specifically. Indeed, available data show that metabolic alterations and endocrine disturbances can specifically and separately influence patients’ clinical evolution (19, 20), whereas cutaneous (e.g., pressure sores) and musculo-skeletal complications are both particularly frequent in patients with sABI but imply tailored treatment and differentially impact on outcomes (21). Moreover, a new category “sepsis,” i.e., “life-threatening organ dysfunction caused by a dysregulated host response to infection” (22) was added, as patients with acute neurologic injury are usually at higher risk for developing in hospital sepsis, due to frequent need for ventilatory support and intravenous therapy via central line catheters that increases the exposition to repeated interactions with multi-resistant pathogens in the lower respiratory trait and in the bloodstream (23). Sepsis has been found to increase mortality in the acute phase of patients with traumatic brain injury (24) and to be the most common cause of in-hospital deaths in several countries (23). Sepsis was diagnosed according to the quick SOFA score criteria [i.e., respiratory rate of 22/min or greater, altered mentation, or systolic blood pressure of 100 mm Hg or less (22)], regardless of the source of infection. Presence of infection of each system/apparatus is reported in their specific categories (3–9), but, for instance, in case of a severe urinary tract infection leading to sepsis, a score is assigned both for the urinary category and for sepsis category.

As for the numeric system for rating clinical complexity of patients with sABI, the FDG-CCS classifies severity of medical complications included in groups 1–9 on a 4-point ordinal scale, with score 0 meaning lack of complication, whereas scores 1–3 are assigned based on the requirement and intensity of the therapeutic intervention: 1 = no treatment required (mild complication), 2 = required pharmacological or non-pharmacological treatment (e.g., dressing for pressure sores) not associated with intensive clinical monitoring (moderate complication), and 3 = required urgent and/or continuous or sub-continuous treatment and clinical monitoring or surgical interventions (severe complication). The 4-point score (i.e., 0–3) is assigned based on frequency and duration of the episodes for categories 10 (epilepsy/myoclonus) and 11 (paroxysmal sympathetic hyperactivity), and as a function of the number of affected joints for category 12 (heterotopic ossifications). Lastly, the category 13 (sepsis) is rated 0 if absent or 1 if present. In case of occurrence of several clinical complications within the same category, only the most severe is scored.

The FDG-CCS total score ranges from 0 (i.e., no complications) to 37 (presence of all clinical complications with the highest severity). A detailed description of the FDG-CCS and the comparison with original categorization are reported in Supplementary material and Table 1.

*In the second phase*, a single online training session was performed with professionals from the six neurorehabilitation units, including several professionals who did not participate in the development of the FDG-CCS. This phase was dedicated to present administration and scoring procedures for the Italian version of the FDG-CCS and the CoCoS. Subsequently, all the participating professionals were required to administer and score FDG-CCS on 3 patients with sABI, whose detailed medical reports and paraclinical evaluations (e.g., laboratory tests, radiological exams) performed in a 15-day hospital stay were provided by the study coordinator. Professionals’ scores were analyzed to detect potential inconsistencies among examiners raters and any difficulty in the administration protocol were discussed. At the end of the training phase an inter-rater agreement was calculated for each of the 3 cases. Inter-rater agreement was poor for the score of a clinical complication in the gastrointestinal category (constipation), as the treatment administered (i.e., evacuation enema) was classified as moderate in half of the examiners and urgent in the other half. In the evaluated patients, the correct score was 2 (moderate severity), as it was sufficient to administer the treatment once, and without need of intensive monitoring. Another issue was raised about the possible difficulty to classify some metabolic alterations as metabolic or endocrine. For instance, hypo- or hyper-glycemia could be classified in metabolic category and as a marker of an endocrine disorder (i.e., diabetes mellitus). However, in case of coexistence of metabolic alterations and endocrine disorders, the FDG-CCS protocol recommended scoring only the clinical sign (e.g., hypo- or hyper-glycemia) if the related endocrine disorder (e.g., diabetes mellitus) has not been diagnosed, otherwise scoring only the endocrine disorder (see Supplementary material).

*In the third phase* of the study, three professionals (A, B, and C) for each participating center independently administered and scored the FDG-CCS (examiners A and B) and the CoCoS (examiner C) in a sample of sABI inpatients, to assess the inter-rater agreement and concurrent validity of the FDG-CCS.

### 2.2 Participants in the validation study

Within each of the six participating intensive neurorehabilitation units, three professionals who were potential or actual caretakers of sABI patients were identified as examiners A, B and C in the validation study. Although physicians have specific expertise on clinical complication management, we also involved non-physician professionals for two main reasons: first, we aimed at proposing a well operationalized tool, with simple and clear scoring criteria to be applied in usual rehabilitation settings; second, in rehabilitation settings non-physician professionals are usually informed about patient’s clinical complications, treatment, rehabilitation program and outcomes during the periodic team meetings, so that they are in the position of contributing to assessment of patients’ clinical complexity. All recruited examiners (*n* = 18) had prior experience in the care of patients with sABI; they worked as physicians (*n* = 13), physiotherapists (*n* = 2), speech therapists (*n* = 2) or nurses (*n* = 1). Each participating center enrolled the first 7 consecutively admitted patients with sABI in a period of 6 months (May–October 2024) and who met the following inclusion criteria: (i) age ≥ 18 years; (ii) clinical diagnosis of sABI (i.e., coma >24 h with Glasgow Coma Scale ≤8 after traumatic, vascular, or anoxic brain injury); (iii) time post-injury ≥28 days; (iv) written informed consent by the patient or by her/his legal representative when the patient could not give the consent him/herself.

Since we aimed at validating a novel clinical tool, we had no reference in the literature for estimating the expected effect size. For this reason, we computed the sample size focusing on the correlation between the FDG-CCS and the CoCoS, assessed by Spearman’s rank correlation coefficient (*ρ*). As we hypothesized the presence of a correlation (i.e., discarding the null hypothesis of ρ ≠ 0), although of small-moderate entity (i.e., *ρ* = 0.50), between the scales, sample size calculation was done assuming as null hypothesis (H0) the absence of correlation (ρ = 0) and as alternative hypothesis (H1) the existence of correlation between the two scales (ρ ≠ 0). The sample was estimated by the G*Power software (25), using a *t*-test for correlation and using a two-tailed test and assuming a correlation between the scales of ρ = 0.5, a type I error of 0.05 and a power of 90%. On these bases, 42 patients were recruited, 20% of them to cope with possible dropouts.

### 2.3 Procedure

*At study entry* (i.e., enrolment), the following patients’ data were collected: i. demographic (age, sex); ii. medical history (time post-injury, etiology, CIRS comorbidity and severity scores prior to the brain injury); iii. Clinical features (diagnosis based on standardized clinical criteria of VS/UWS, MCS *minus*, MCS *plus*, or emergence from DoC) (5, 26); consciousness level measured by the best total score (out of at least three in a week) of the Italian version of the Coma Recovery Scale-Revised (CRS-R) (27); functional level assessed by means of the Glasgow Outcome Scale-Extended score (GOS-E) (28); level of cognitive functioning score (LCF) (29); disability level assessed by the Disability Rating Scale total score (DRS) (30).

*On the 15th day from enrolment*, two independent examiners (examiners A and B) administered the FDG-CCS taking into account the previous 2 weeks as observation time. This procedure was implemented in order to level out the probability of occurrence of clinical complications among patients enrolled from the different centers, which could be influenced by time post-injury and by duration of hospitalization. For the purposes of the study, the examiners scored both novel incident complications and acute relapses or worsening of pre-existing medical comorbidities if they required moderate (i.e., pharmacological or non-pharmacological treatment but not intensive clinical monitoring) or intensive (i.e., urgent with a continuous or sub-continuous clinical monitoring) treatment during the two-week observation period. Concurrently, a third examiner (examiner C), blind to the score of the FDG-CCS of A and B examiners, administered the CoCoS on the same observation period. The 3 examiners scored the items of the FDG-CCS and of the CoCoS only based on information documented in the medical records, by laboratory tests and/or instrumental examinations or by direct clinical observation.

### 2.4 CoCoS

The CoCoS is a measurement tool designed specifically to assess comorbidities in patients with pDoC on recovery (17). The CoCoS consists of 24 categories addressing the frequency of various comorbidities common in this subset of patients. The categories include respiratory and urinary tract infections, non-infectious respiratory disorders, structural heart diseases, rhythm disorders without structural heart diseases, arterial hypertension, diabetes mellitus, dysautonomia, peripheral artery or venous diseases, with a specific separated category for those of supra-aortic trunks, hepatobiliary and gastrointestinal disorders, seizures, hydrocephalus, fractures and joint diseases, anemia, presence of life support devices, pressure ulcers, malignancies, malnutrition, renal diseases, and previous disability. For each category, scoring is based on the presence/absence of the comorbidity and its severity. Severity is scored based on presence of symptoms, the need for treatment and the response to it. The severity due to the need of life support devices is scored considering the number of necessary devices (tracheostomy tube, nasogastric tube, percutaneous endoscopic gastrostomy, urinary catheter, central venous catheter). The cumulative comorbidity burden is denoted by the final summative score ranging from 0 (no comorbidities) to 72 (presence of all comorbidities at maximum of their severity). Based on the final cumulative score, patients are stratified in 3 severity groups using the following cut-off scores: (0: no comorbidities; range 1–24: presence of mild comorbidities; range 25–48: moderate comorbidities; range 49–72: severe comorbidities).

### 2.5 Data management

The investigators in charge of data collection also took care of the data entry. Patients’ data were collected and entered in a pseudonymised form into a centralized, password-protected, electronic database on REDCap (Research Electronic Data Capture), following as much as possible a forced-choice format. REDCap complies with the Health Insurance Portability and Accountability Act in compliance with security measures and therefore only contains data that has already been pseudonymised. Indeed, the personal data of each patient were de-identified and replaced by an alphanumeric ID code in an ‘association key’ document accessible exclusively to the enrolling center in a password-protected Excel file and then entered into REDCap. Moreover, REDCap ensures blindness between two examiners, as each examiner is not allowed to see the other’s assessment.

### 2.6 Statistical analyses

Demographic and clinical data were submitted to Shapiro–Wilk tests to investigate the normality of the distributions. As these preliminary analyses showed that not all continuous variables followed a normal distribution, except for age, the descriptive data of the sample on admission to the study were expressed in terms of mean ± standard deviation for age, median (inter-quartile range, IQR) for all other continuous variables, and as frequencies for categorical variables. Parametric or non-parametric analyses were performed accordingly. In particular, demographic and clinical variables were compared between conscious and pDoC patients, and between VS/UWS and MCS patients, by means of *t*-test, the Mann–Whitney U test or χ^2^-test, as appropriate.

The FDG-CCS total score of both examiner A and B was compared between conscious and pDoC patients, and between VS/UWS and MCS patients by means of Mann–Whitney U tests.

For the assessment of inter-rater agreement in the administration of the FDG-CCS, Cohen’s K was calculated on the scores of the individual sub-scales and the intra-class coefficient (ICC) was calculated on the total scores of the FDG-CCS, administered by the two examiners (A and B). For Cohen’s K, the values of 0.4 or less can be considered poor, values between 0.4 and 0.6 moderate, values between 0.6 and 0.8 good, whereas values greater than 0.8 suggest excellent inter-observer agreement (31). For ICC, values less than 0.5 are indicative of poor reliability, values between 0.5 and 0.75 indicate moderate reliability, values between 0.75 and 0.9 indicate good reliability, and values greater than 0.90 indicate excellent reliability (32).

For the test of concurrent validity between the FDG-CCS (administered by both examiners A and B) and the CoCoS (administered by examiner C), Spearman’s rank correlation coefficient was calculated on the total scores obtained by the patients on the two scales.

The results were considered statistically significant if *p* < 0.05. All analyses were performed using IBM SPSS v.25 (IBM Corp., Armonk, NY, United States).

## 3 Results

### 3.1 Description of the sample

Forty-two patients with sABI were enrolled in the study, including 22 patients with pDoC and 20 fully conscious patients at study entry. Conscious patients and patients with pDoC did not differ in terms of age, sex, etiology, CIRS severity and comorbidity scores, but significantly differed in terms of time post-injury, clinical diagnosis, CRS-R, LCF, GOS-E, and DRS scores. Table 2 presents the demographic and clinical data of the participants, categorized by clinical diagnosis of consciousness. Within the pDoC group, patients in VS/UWS did not differ from those in MCS in terms of demographic or anamnestic characteristics (all *p* > 0.05), but the two diagnostic sub-groups differ significantly in the CRS-R [U = 2.5; *p* > 0.005; VS/UWS median = 6 (IQR = 2) vs. MCS = 12 (4)], LCF [U = 10.5; *p* = 0.001; VS/UWS = 2 (0) vs. MCS = 3 (9)], GOS-E [U = 28.5; *p* = 0.043; VS/UWS = 2 (0) vs. MCS = 3 (1)], and DRS [U = 25.0; *p* = 0.025; VS/UWS = 24 (0) vs. MCS = 22 (3)] scores.

**Table 2:** Patients’ demographic and clinical characteristics at study entry, as a function of clinical diagnosis.

	Overall	pDoC	Conscious	
	*n* = 42	*n* = 22	*n* = 20	*t/U/*χ^2^ (*p*)
Age [years; mean ± SD]	58.8 ± 15.6	60.2 ± 16.0	57.2 ± 15.3	0.604 (0.550)
Sex [F/M; *n* (%)]	19/23	11/11		0.423 (0.516)
TPI [months; median (IQR)]	2.7 (3.5)	3.3 (7.0)	2.1 (2.1)	133.0 (0**.028**)
Etiology [*n* (%)]				5.334 (0.502)
TBI	8	3	5	
Hemorrhagic stroke	16	9	7	
Ischemic stroke	3	1	2	
Anoxic	8	6	2	
Mixed	5	2	3	
Other	2	1	1	
CIRS severity [median (IQR)]	1.1 (0.3)	1.1 (0.3)	1.1 (0.3)	188.0 (0.414)
CIRS comorbidity [median (IQR)]	1 (2)	1 (2)	0.5 (1)	183.0 (0.324)
Diagnosis [*n* (%)]				42.0 (**<0.005**)
VS/UWS	13	13	0	
MCS *minus*	7	7	0	
MCS *plus*	2	2	0	
Conscious	20	0	20	
CRS-R [median (IQR)]	13 (17)	7.5 (5)	23 (2)	18.5 (**<0.005**)
LCF [median (IQR)]	3.5 (3)	2 (1)	5 (2)	3.5 (**<0.005**)
GOS-E [median (IQR)]	3 (1)	2 (1)	3 (0)	76.0 (**<0.005**)
DRS [median (IQR)]	21.5 (6)	24 (1)	18 (3)	9.0 (**<0.005**)

Descriptive data are reported as mean ± SD or median (inter-quartile range), as appropriate, for continuous variables and as counts (percentage) for categorical variables. Univariate statistics are based upon the *t*-test, the Mann–Whitney U test or χ^2^-test, as appropriate. Significant *p*-values are printed in bold. CIRS, Cumulative Illness Rating Scale; CRS-R, Coma Recovery Scale-Revised; DRS, Disability Rating Scale; F, Female; GOS-E, Glasgow Outcome Scale-Extended; LCF, Level of Cognitive Functioning; M, Male; MCS, Minimally Conscious State; pDoC, prolonged Disorders of Consciousness; SD, Standard Deviation; TBI, Traumatic Brain Injury; TPI, Time Post-Injury; UWS, Unresponsive Wakefulness Syndrome; VS, Vegetative State.

### 3.2 Distribution of frequency and severity of clinical complications

**Figure 1:**
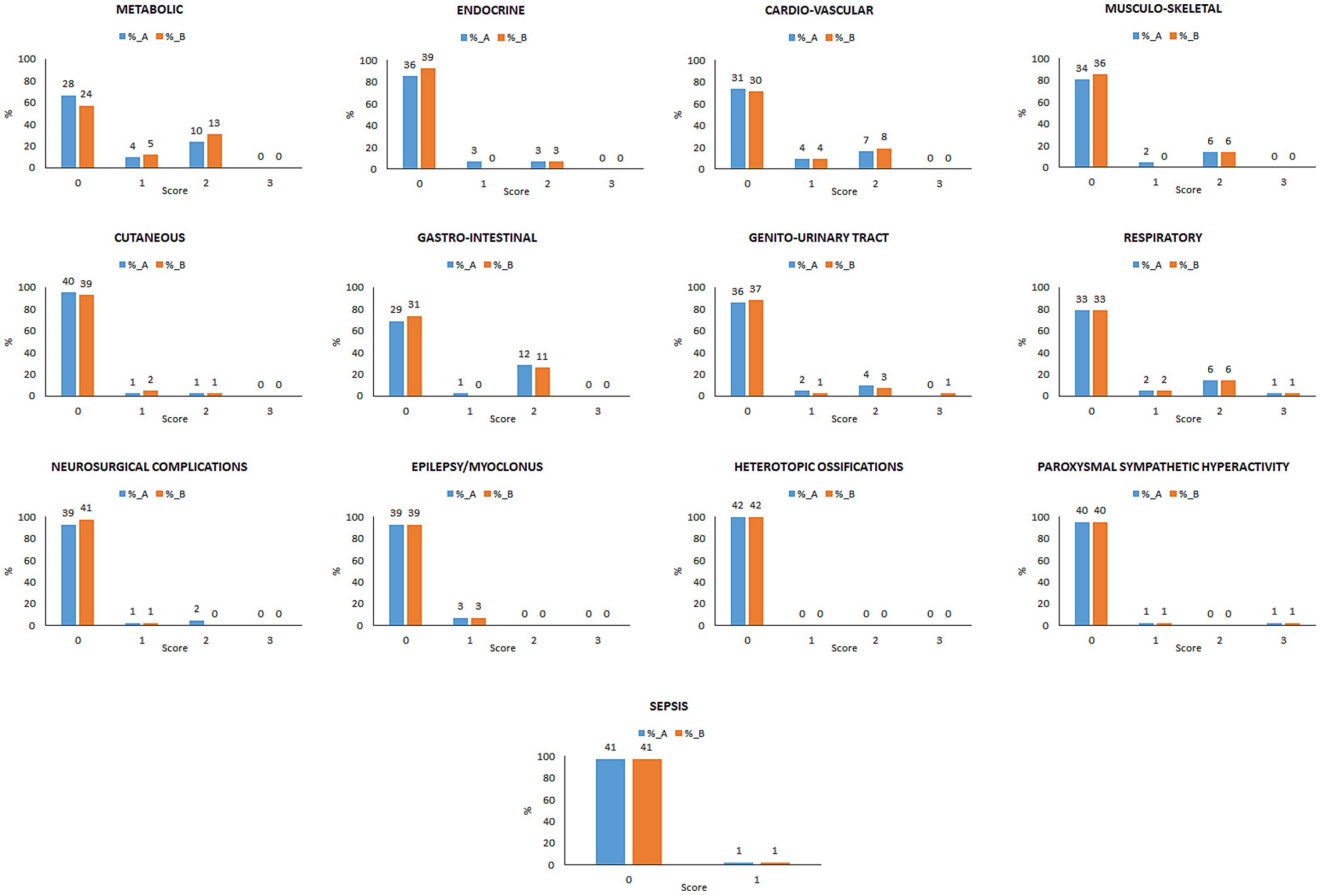
Frequency and severity distribution of clinical complications evaluated by examiners A and B by means of the FDG-CCS in the overall validation sample.

The frequency and severity distribution of clinical complications evaluated by the FDG-CCS, as assessed by both examiners A and B, is shown in Figure 1. The median total score for examiners A was 3 (IQR = 5), whereas for examiner B was 3 (IQR = 4). The FDG-CCS total score did not differ between conscious and pDoC patients, nor between VS/UWS and MCS, for both examiners A and B (all *p* > 0.05).

Metabolic (Examiner A = 33%; Examiner B = 43%), gastro-intestinal (A = 31%; B = 26%), cardio-vascular (A = 26%; B = 29%), respiratory (A = 21%; B = 21%), and musculo-skeletal disorders (A = 19%; B = 14%) were the most frequent complications; sepsis was reported in 2% of the cases.

The only complications that were rated as severe by both examiners A and B were respiratory and paroxysmal sympathetic hyperactivity, in 5% (*n* = 2/42) of the cases. On average, the complications were rated as mild in 4.3% (mean percentage between examiners) of the cases, and moderate in the 9.9% of the cases. The complications that were rated as moderate more frequently were metabolic (27%) and gastro-intestinal (27%).

Metabolic complications were more frequent in conscious (Examiner A = 45%; Examiner B = 65%) than in pDoC patients (A = 22.7%; B = 22.7%; A-χ^2^ = 11.617; A-*p* = 0.003; B-χ^2^ = 10.526; B-*p* = 0.005). No significant differences in frequency or severity of any FDG-CCS category have been observed between VS/UWS and MCS (all *p* > 0.05).

### 3.3 Inter-rater agreement and concurrent validity

Inter-rater agreement for the total score of the FDG-CCS was good. Inter-rater agreement for single FDG-CCS categories was excellent for cardio-vascular, musculo-skeletal, gastro-intestinal, epilepsy/myoclonus, heterotopic ossification, paroxysmal sympathetic hyperactivity, and sepsis; good for cutaneous and respiratory complications; moderate for endocrine, genito-urinary tract, and neurosurgical complications; poor for metabolic complications (see Table 3 for detailed values).

**Table 3:** Inter-rater agreement for each category’s scores and total score of the FDG-CCS.

	K/ICC	*p*
Metabolic	0.376	**0.002**
Endocrine	0.521	**<0.005**
Cardio-vascular	0.890	**<0.005**
Musculo-skeletal	0.833	**<0.005**
Cutaneous	0.791	**<0.005**
Gastro-intestinal	0.885	**<0.005**
Genito-urinary tract	0.598	**<0.005**
Respiratory	0.669	**<0.005**
Neurosurgical complications	0.488	**<0.005**
Epilepsy/Myoclonus	1.0	**<0.005**
Heterotopic ossifications	1.0	**<0.005**
Paroxysmal sympathetic hyperactivity	1.0	**<0.005**
Sepsis	1.0	**<0.005**
Total score	0.865	**<0.005**

Inter-rater agreement is expressed as Cohen’s K for single categories and as ICC for the total score. Significant p-values are printed in bold. For Cohen’s K, the values of 0.4 or less can be considered poor, values between 0.4 and 0.6 moderate, values between 0.6 and 0.8 good, whereas values greater than 0.8 suggest excellent inter-observer agreement. For ICC, values less than 0.5 are indicative of poor reliability, values between 0.5 and 0.75 indicate moderate reliability, values between 0.75 and 0.9 indicate good reliability, and values greater than 0.90 indicate excellent reliability. CI, Confidence Intervals; FDG-CCS, Fondazione Don Gnocchi-Clinical Complication Scale; ICC, Intraclass Correlation Coefficient.

Physicians (*n* = 13) who scored the FDG-CCS items as examiners A and B showed lower inter-rater agreement with respect to non-physicians (*n* = 4; i.e., 1 physiotherapist, 2 speech-therapists, 1 nurse). In particular, non-physicians provided an excellent inter-rater agreement for endocrine, cardio-vascular, musculo-skeletal, cutaneous, gastro-intestinal, respiratory, neurosurgical, epilepsy/myoclonus, heterotopic ossification, paroxysmal sympathetic hyperactivity, and sepsis (all K = 1.00; *p* < 0.005); good for genito-urinary tract (K = 0.725; *p* < 0.005); moderate for metabolic complications (K = 0.481; *p* < 0.005); and an excellent reliability for the total score (ICC = 0.989; *p* < 0.005).

Conversely, physicians provided an excellent inter-rater agreement for cardio-vascular (K = 0.873; *p* < 0.005), gastro-intestinal (K = 0.859; *p* < 0.005), epilepsy/myoclonus, heterotopic ossification, paroxysmal sympathetic hyperactivity, and sepsis (all K = 1.00; *p* < 0.005); good for musculo-skeletal (K = 0.797; *p* < 0.005), cutaneous (K = 0.650; *p* < 0.005), and respiratory complications (K = 0.605; *p* < 0.005); moderate for genito-urinary tract (K = 0.520; *p* = 0.002) and neurosurgical complications (K = 0.481; *p* < 0.005); poor for metabolic (K = 0.251; *p* = 0.070) and endocrine complications (K = 0.385; *p* = 0.001); and a good reliability for the total score (ICC = 0.773; *p* < 0.005).

As for concurrent validity, the median total score of the CoCoS was 8 (IQR = 8). The FDG-CCS total score correlated significantly with the total score of the CoCoS, for both the examiner A (*ρ* = 0.356; *p* = 0.01) and B (ρ = 0.317; *p* = 0.02).

## 4 Discussion

The present study proposed a novel clinical tool, the FDG-CCS, for keeping track of clinical complications and their severity in patients with sABI. The FDG-CCS has been developed as an update of an original preliminary checklist of clinical complications, devised to evaluate their impact on long-term evolution in a large cohort of patients with pDoC (6). The FDG-CCS differed from the original checklist in the total number of categories (13 instead of 10) as 2 original items have been split in 4 different groups of clinical complications, and sepsis has been added as a new category, to specifically investigate their occurrence and impact on patient’s outcome (23, 24). Additionally, the FDG-CCS numerically rated the presence and the severity level of each category, in order to provide clinicians with a validated tool to classify patients’ clinical complications, and to monitor their clinical evolution or response to the treatment.

In our cohort of patients, the frequency and severity of complications identified by means of the FDG-CCS did not differ between patients with pDoC and those who emerged from DoC. This apparently counterintuitive finding could be explained by the fact that many clinical complications (e.g., respiratory infections or neurogenic heterotopic ossification) (11) are related to long-lasting immobility and disability (33), which in our cohort of patients ranges from “extreme vegetative state, DRS = 27” in pDoC to “extremely severe disability, DRS = 18” in those emerged from DoC. Further cross-sectional studies on large cohorts of patients are needed for evaluating the ability of the FDG-CCS to identify different levels of clinical complexity in patients with different levels of consciousness.

The present validation study showed a good inter-rater agreement of the FDG-CCS total score and excellent or good inter-rater agreement in the majority of categories and moderate in only three groups (i.e., endocrine, genito-urinary, and neurosurgical complications). This finding seems to support the idea that the FDG-CCS can be used in clinical practice. However, metabolic complications had a poor inter-rater agreement. The poor agreement could be ascribed to the fact that metabolic complications include a wide range of possible alterations in the homeostasis (e.g., hypo- or hypernatremia, hypo- or hyper-glycemia) frequently related to other clinical complications. For this reason, this field is highly heterogeneous and therefore more prone to different interpretations among clinicians as a single clinical complication. Furthermore, although highly prevalent in sABI, the metabolic disorders may not require specific attention and treatment unless the alterations are particularly severe. As a result, mild metabolic alterations are often underestimated or go undiagnosed, contributing to variability of clinical interpretation in the FDG-CCS. Additionally, scoring of severity level based on the need for treatment (especially non-intensive treatment) may have led to discrepancies among examiners given the lack of standardized treatment protocols for some metabolic disorders (e.g., mild anemia in patients with artificial enteral nutrition). However, early detection of metabolic imbalances is strongly recommended, as they can impact the length of hospital stay and patient mortality in both brain injury patients (6) as well as in the general inpatient population (34). Moreover, some of them are related to endocrine alterations (e.g., hyponatremia due to syndrome of inappropriate antidiuretic hormone secretion) that have been found to prolong hospital stay in traumatic brain injury (35). Although metabolic alterations were not scored consistently by different examiners in our cohort of patients with sABI, they were the most frequent complications, along with gastro-intestinal, cardio-vascular, respiratory, and musculo-skeletal complications. These findings are consistent with the previous monocentric and multicenter studies on patients with pDoC (6) and in an overall cohort of patients with sABI (1).

Additionally, we investigated whether the low agreement on certain categories could be influenced by the type of examiner (medical or non-medical). Surprisingly, we found greater disagreement in physicians than in non-physicians. This finding could be likely attributed to the fact that the physicians’ scores could be influenced by the current open debates about treatment of certain clinical complications. For example, there is no definitive consensus on the hemoglobin level for the treatment of anemia in chronic stable disease (36) or on criteria for the treatment of asymptomatic bacteriuria in special populations of patients at high risk of catheter-related urinary tract infections or neurogenic bladder infections (37, 38). The discrepancies between the two professional groups (physicians and non-physicians) warrant to be addressed in larger samples of participants.

Lastly, the total score of FDG-CCS and CoCoS correlated significantly. However, the concurrent validity between the two scales was not very high. This observation could be explained by the differences in scoring systems and categorization of clinical complications of the two scales. Although very detailed, the CoCoS does not allow for recording dysfunctions frequent in the populations of patients with sABI, such as heterotopic ossifications or spasticity. Moreover, severity classification in CoCoS is based on criteria that differ for each disease category (e.g., anemia severity is quantified based on hemoglobin levels and need for transfusion; diabetes based on glycaemic index), which makes it challenging to compile the scale during routine clinical practice. The FDG-CCS aims to overcome these pitfalls, as it assesses clinical complications that are specific to (e.g., paroxysmal sympathetic hyperactivity) (39) or more frequent in patients with sABI (e.g., heterotopic ossifications) (11).

This study has some limitations. Firstly, we did not evaluate clinical complications that significantly affect survival and consciousness recovery (40) in the acute phase, and this might limit the generalization of the results of this study. Secondly, we did not evaluate the ability of FDG-CCS total score to profile patients’ clinical complexity, because of the small number of patients in the two diagnostic subgroups (i.e., with and emerged from pDoC). Thirdly, we did not consider all clinical complications that occurred from the admission to the rehabilitation unit, as for the sake of experimental control we scored the FDG-CCS referencing a 15-day period. Indeed, the main purpose of our study was to develop and validate a specific tool for categorizing clinical complications in a specific observation time window and not to evaluate the overall occurrence of the clinical complications that affect this population since the brain injury. Further multicenter studies on a larger cohort of patients with sABI, including patients admitted to intensive care and post-acute rehabilitation, could confirm the ability of the FDG-CCS to profile patients’ clinical complexity. Also, longitudinal studies could assess the possible prognostic value of the FDG-CCS as it can record clinical complications that can impact on patients’ outcomes (6, 41). Lastly, our sample size was not sufficient to perform a factor analysis that would have allowed us to explore any latent factors explaining variance of the scale.

Notwithstanding the above limitations, the present study proposed and validated a novel clinical tool for the categorization of clinical complications of patients with sABI. This tool showed sufficient inter-rater agreement among different rehabilitation centers, thus allowing the rehabilitation team to administer it for the categorization of clinical complications and their severity in routine clinical practice. The FDG-CCS could help rehabilitation teams to plan tailored treatments on the basis of patients’ clinical severity and complexity, as accurate detection of some clinical complications can guide rehabilitation programs. For instance, the presence of musculo-skeletal complications or heterotopic ossifications are contraindications for verticalization (42). Indeed, in the cohort of patients enrolled for the validation study, we found a relatively high occurrence of clinical complications, some of which were more frequent than in previous observational studies (1, 6). These findings confirm that individuals with sABI are medically complex and require clinical monitoring by an experienced multidisciplinary team to prevent and manage their clinical problems.

## Data Availability

The raw data supporting the conclusions of this article will be made available by the authors, without undue reservation.

## Footnotes

^1^ https://www.dongnocchi.it/@servizi/dipartimento-gca
