# Supplementary material for: The clinical complication scale of Fondazione Don Gnocchi for classifying clinical complications in patients with severe acquired brain injury: development and multicenter validation
Source: Front Neurol. 2025 Mar 12;16:1537093. doi: 10.3389/fneur.2025.1537093 (PMC11936823; doi:10.3389/fneur.2025.1537093)
Supplement: Supplementary file 1 [file Data_Sheet_1.docx]

***Supplementary Material***

**Italian version of the Clinical Complication Scale of Fondazione Don Gnocchi (FDG-CCS), as used in the validation study.**

| **COMPLICANZA CLINICA** | **Tipo di complicanza**  **(inserire tutte le complicanze presenti per ciascuna categoria indipendentemente dalla gravità)** | **Punteggio**  **(inserire il punteggio della**  **complicanza più grave per ciascuna categoria)** | |
| --- | --- | --- | --- |
|  |  |  |  |
| 1. **Anomalie metaboliche*** | Squilibri elettrolitici, anemia, ipoalbuminemia, malnutrizione, altro | **ASSENTE** | **0** |
|  |  | **LIEVE** | **1** |
|  |  | **MODERATO** | **2** |
|  |  | **GRAVE** | **3** |
| 1. **Disturbi endocrinologici*** | distiroidismi, diabete mellito, diabete insipido, SIADH, altro | **ASSENTE** | **0** |
|  |  | **LIEVE** | **1** |
|  |  | **MODERATO** | **2** |
|  |  | **GRAVE** | **3** |
| 1. **Disturbi cardio-vascolari*** | Insufficienza cardiaca da infarto miocardico acuto, insufficienza cardiaca congestizia, aritmia acuta o cronica, trombosi artero-venosa, altro | **ASSENTE** | **0** |
|  |  | **LIEVE** | **1** |
|  |  | **MODERATO** | **2** |
|  |  | **GRAVE** | **3** |
| 1. **Problemi muscolo-scheletrici*** | Spasticità, fratture, lesioni muscolari, lesioni tendinee, retrazioni tendinee, altro | **ASSENTE** | **0** |
|  |  | **LIEVE** | **1** |
|  |  | **MODERATO** | **2** |
|  |  | **GRAVE** | **3** |
| 1. **Problemi cutanei*** | piaghe da decubito, ferite, accessi cutanei, altro | **ASSENTE** | **0** |
|  |  | **LIEVE** | **1** |
|  |  | **MODERATO** | **2** |
|  |  | **GRAVE** | **3** |
| 1. **Disturbi gastro-intestinali*** | Emorragie, ulcera, occlusione o paralisi intestinale, peritonite, enterite da Clostridium difficile, diarrea di ndd, infarto intestinale, colelitiasi, epatite, pancreatiti, altro | **ASSENTE** | **0** |
|  |  | **LIEVE** | **1** |
|  |  | **MODERATO** | **2** |
|  |  | **GRAVE** | **3** |
| 1. **Disturbi genito-urinari*** | Infezioni, emorragie, calcoli urinari, ostruzioni delle vie urinarie, insufficienza renale acuta o cronica, altri | **ASSENTE** | **0** |
|  |  | **LIEVE** | **1** |
|  |  | **MODERATO** | **2** |
|  |  | **GRAVE** | **3** |
| 1. **Disturbi respiratori*** | Polmoniti, BPCO, insufficienza respiratoria, tracheomalacia, stenosi tracheale, fistola tracheo-esofagea, altro | **ASSENTE** | **0** |
|  |  | **LIEVE** | **1** |
|  |  | **MODERATO** | **2** |
|  |  | **GRAVE** | **3** |
| 1. **Complicanze neurochirurgiche*** | Idrocefalo, nuova lesione cerebrale, disfunzioni della derivazione ventricolo-peritoneale, altro | **ASSENTE** | **0** |
|  |  | **LIEVE** | **1** |
|  |  | **MODERATO** | **2** |
|  |  | **GRAVE** | **3** |
| 1. **Epilessia o mioclono**** | Crisi epilettiche generalizzate o parziali, mioclono generalizzato o parziale, stato epilettico convulsivo, stato epilettico non convulsivo, altro | **ASSENTE** | **0** |
|  |  | **LIEVE** | **1** |
|  |  | **MODERATO** | **2** |
|  |  | **GRAVE** | **3** |
| 1. **Calcificazioni eterotopiche*****   (ecograficamente o radiologicamente evidenti) | Specificare sedi coinvolte | **ASSENTE** | **0** |
|  |  | **LIEVE** | **1** |
|  |  | **MODERATO** | **2** |
|  |  | **GRAVE** | **3** |
| 1. **Crisi neurovegetative******   Episodi parossistici di iperattività simpatica (tachicardia, tachipnea, diaforesi, posture anomale, iperpiressia) (1) | Specificare semeiologia | **ASSENTE** | **0** |
|  |  | **SPORADICO** | **1** |
|  |  | **FREQUENTE** | **2** |
|  |  | **PERSISTENTE** | **3** |
| 1. **Sepsi*******   Disfunzione d'organo pericolosa per la vita causata da una risposta disregolata dell'ospite all'infezione (2).  Infezione + almeno 2 quickSOFA:   - PAS ≤ 100 mmHg - GCS <15 - Freq. resp. ≥ 22/min | Specificare apparato/organo coinvolto (se identificabile) | **ASSENTE** | **0** |
|  |  | **PRESENTE** | **1** |
| **TOTALE:**  **RANGE 0-37** | |  | |

*** LIVELLO DI GRAVITA’**

0. ASSENTE

1. LIEVE: non richiedono interventi terapeutici, possono interferire con la clinica

2. MODERATA: richiedono interventi terapeutici, in quanto interferiscono significativamente con la clinica, ma non richiedono monitoraggio clinico intensivo

3. GRAVE: richiedono interventi terapeutici intensivi (urgenti e/o continui/subcontinui), in quanto potenzialmente letali, e che richiedono monitoraggio clinico intensivo

**** LIVELLO DI GRAVITA’ (epilessia o mioclono)**

0. ASSENTE

1. LIEVE: presenza di un episodio

2. MODERATA: più di un episodio ma controllati da terapia

3. GRAVE: crisi o mioclonie frequenti o subcontinue resistente a farmaci o stato epilettico convulsivo o non convulsivo

***** LIVELLO DI GRAVITA’ (calcificazioni eterotopiche)**

0. ASSENTE

1. LIEVE: una localizzazione

2. MODERATA: localizzazioni multiple

3. GRAVE: postura coatta dell’arto coinvolto non riducibile

****** FREQUENZA (crisi neurovegetative)**

0. ASSENTE

1. SPORADICO: episodi per un periodo <3 giorni

2. FREQUENTE: episodi per un periodo >3 giorni e <2 settimane

3. PERSISTENTE: episodi per ≥2 settimane

******* PRESENZA (sepsi)**

0. ASSENTE

1. PRESENTE

*Note*: In caso di coesistenza di alterazioni metaboliche e disturbi endocrini, si raccomanda di assegnare un punteggio solo al segno clinico (per esempio, ipo- o iper-glicemia) come alterazione metabolica se il disturbo endocrino correlato (per esempio, diabete mellito) non è stato diagnosticato, altrimenti assegnare un punteggio solo alla malattia endocrina.

*Abbreviazioni:* BPCO = Broncopneumopatia Cronica Ostruttiva; Freq. resp.= Frequenza respiratoria; GCS = Glasgow Coma Scale; SIADH = Sindrome da inappropriata secrezione di ormone antidiuretico; mmHg = millimetri di mercurio; PAS = Pressione Arteriosa Sistolica; SOFA = Sequential Organ Failure Assessment.

**English version of the Clinical Complication Scale of Fondazione Don Gnocchi (FDG-CCS).**

| **CLINICAL COMPLICATION** | **Specific complication**  **(enter all complications present for each category regardless of severity)** | **Score**  **(enter the score of the**  **most serious complication for each category)** | |
| --- | --- | --- | --- |
|  |  |  |  |
| 1. **Metabolic*** | Electrolyte imbalances, anaemia, hypoalbuminemia, malnutrition, other | **ABSENT** | **0** |
|  |  | **MILD** | **1** |
|  |  | **MODERATE** | **2** |
|  |  | **SEVERE** | **3** |
| 1. **Endocrine*** | Dysthyroidism, hypopituitarism, diabetes mellitus, diabetes insipidus, SIADH, central salt wasting syndrome, adrenocortical insufficiency, other | **ABSENT** | **0** |
|  |  | **MILD** | **1** |
|  |  | **MODERATE** | **2** |
|  |  | **SEVERE** | **3** |
| 1. **Cardio-vascular*** | Heart failure with preserved or reduced ejection fraction, acute or chronic myocardial ischaemia, acute or chronic arrhythmia, arteriovenous thrombosis, other | **ABSENT** | **0** |
|  |  | **MILD** | **1** |
|  |  | **MODERATE** | **2** |
|  |  | **SEVERE** | **3** |
| 1. **Musculo-skeletal*** | Spasticity, fractures, muscle injuries, tendon injuries, tendon retractions, other | **ABSENT** | **0** |
|  |  | **MILD** | **1** |
|  |  | **MODERATE** | **2** |
|  |  | **SEVERE** | **3** |
| 1. **Cutaneous*** | Bed sores, wounds, skin accesses, other | **ABSENT** | **0** |
|  |  | **MILD** | **1** |
|  |  | **MODERATE** | **2** |
|  |  | **SEVERE** | **3** |
| 1. **Gastro-intestinal*** | Bleeding, ulcer, intestinal obstruction or paralysis, peritonitis, clostridium difficile enteritis, non-specific diarrhoea, intestinal infarction, cholelithiasis, hepatitis, pancreatitis, other | **ABSENT** | **0** |
|  |  | **MILD** | **1** |
|  |  | **MODERATE** | **2** |
|  |  | **SEVERE** | **3** |
| 1. **Genito-urinary tract*** | Infections, bleeding, urolithiasis, urinary tract obstructions, acute or chronic renal failure, other | **ABSENT** | **0** |
|  |  | **MILD** | **1** |
|  |  | **MODERATE** | **2** |
|  |  | **SEVERE** | **3** |
| 1. **Respiratory*** | Pneumonia, COPD, respiratory insufficiency, tracheomalacia, tracheal stenosis, tracheo-oesophageal fistula, other | **ABSENT** | **0** |
|  |  | **MILD** | **1** |
|  |  | **MODERATE** | **2** |
|  |  | **SEVERE** | **3** |
| 1. **Neurosurgical complications*** | Hydrocephalus, new brain injury, ventriculo-peritoneal shunt dysfunction, sinking skin flap syndrome, other | **ABSENT** | **0** |
|  |  | **MILD** | **1** |
|  |  | **MODERATE** | **2** |
|  |  | **SEVERE** | **3** |
| 1. **Epilepsy/myoclonus**** | Generalised or partial seizures, generalised or partial myoclonus, convulsive status epilepticus, non-convulsive status epilepticus, other | **ABSENT** | **0** |
|  |  | **MILD** | **1** |
|  |  | **MODERATE** | **2** |
|  |  | **SEVERE** | **3** |
| 1. **Heterotopic ossifications*****   Paraosteoarthropathies: echographically or radiologically evident heterotopic ossifications | Specify location involved | **ABSENT** | **0** |
|  |  | **MILD** | **1** |
|  |  | **MODERATE** | **2** |
|  |  | **SEVERE** | **3** |
| 1. **Paroxysmal sympathetic hyperactivity******   Paroxysmal episodes of sympathetic hyperactivity (tachycardia, tachypnoea, diaphoresis, abnormal postures, hyperpyrexia) (1) | Specify semiology | **ABSENT** | **0** |
|  |  | **OCCASIONAL** | **1** |
|  |  | **FREQUENT** | **2** |
|  |  | **PERSISTENT** | **3** |
| 1. **Sepsis*******   Life-threatening organ dysfunction caused by a dysregulated host response to infection (2).  Infection + at least 2 quickSOFA   - SBP ≤ 100 mmHg - GCS <15 - Resp. rate ≥ 22/min | Specify system/apparatus or organ of origin (if identified) | **ABSENT** | **0** |
|  |  | **PRESENT** | **1** |
| **TOTAL:**  **RANGE 0-37** | |  | |

*** LEVEL OF SEVERITY**

0. ABSENT

1. MILD: do not require therapeutic interventions, may interfere with clinics

2. MODERATE: require therapeutic interventions, as they significantly interfere with the clinics, but do not require intensive clinical monitoring

3. SEVERE: require intensive therapeutic interventions (urgent and/or continuous/sub-continuous), as they could be life-threatening, and require intensive clinical monitoring

**** LEVEL OF SEVERITY (epilepsy/myoclonus)**

0. ABSENT

1. MILD: presence of one episode

2. MODERATE: more than one episode but controlled by therapy

3. SEVERE: frequent or sub-continuous drug-resistant seizures or myoclonus or convulsive or non-convulsive status epilepticus

***** LEVEL OF SEVERITY (heterotopic ossification)**

0. ABSENT

1. MILD: one location

2. MODERATE: multiple locations

3. SEVERE: coercive posture of the involved non-reducible limb

****** FREQUENCY (paroxysmal sympathetic hyperactivity)**

0. ABSENT

1. OCCASIONAL: episodes lasting <3 days

2. FREQUENT: episodes lasting >3 days and <2 weeks

3. PERSISTENT: episodes lasting ≥2 weeks

******* PRESENCE (sepsis)**

0. ABSENT

1. PRESENT

*Notes*: In case of coexistence of metabolic alterations and endocrine disorder it is recommended scoring only the clinical sign (e.g., hypo- or hyperglycemia) if the related endocrine disorder (e.g., diabetes mellitus) has not been diagnosed, otherwise scoring only the endocrine disorder.

*Abbreviations:* COPD = Chronic Obstructive Pulmonary Disease; GCS = Glasgow Coma Scale; mmHg = millimeters of mercury; Resp. = Respiratory; SIADH = Syndrome of Inappropriate secretion of AntiDiuretic Hormone; SOFA = Sequential Organ Failure Assessment; SPB = Systolic Blood Pressure.

**References:**

1. Baguley IJ, Perkes IE, Fernandez-Ortega JF, et al. Paroxysmal sympathetic hyperactivity after acquired brain injury: consensus on conceptual definition, nomenclature, and diagnostic criteria. J Neurotrauma (2014) 31(17):1515-1520. doi:10.1089/neu.2013.3301
2. Singer M, Deutschman CS, Seymour CW, et al. The Third International Consensus Definitions for Sepsis and Septic Shock (Sepsis-3). JAMA (2016) 315(8):801-810. doi:10.1001/jama.2016.0287
